# Dual Effects of Cyclooxygenase Inhibitors in Combination With CD19.CAR-T Cell Immunotherapy

**DOI:** 10.3389/fimmu.2021.670088

**Published:** 2021-05-26

**Authors:** Mingya Yang, Lei Wang, Ming Ni, Brigitte Neuber, Sanmei Wang, Wenjie Gong, Tim Sauer, Maria-Luisa Schubert, Angela Hückelhoven-Krauss, Ruixiang Xia, Jian Ge, Christian Kleist, Volker Eckstein, Leopold Sellner, Carsten Müller-Tidow, Peter Dreger, Michael Schmitt, Anita Schmitt

**Affiliations:** ^1^ Department of Internal Medicine V, University Clinic Heidelberg, Heidelberg University, Heidelberg, Germany; ^2^ Department of Hematology, the First Affiliated Hospital of Anhui Medical University, Anhui, China; ^3^ Department of Hematology, the Affiliated Hospital of Guizhou Medical University, Guizhou, China; ^4^ Department of Hematology, the first Affiliated Hospital of Soochow University, Suzhou, China; ^5^ Department of Nuclear Medicine, University Clinic Heidelberg, Heidelberg University, Heidelberg, Germany; ^6^ National Center for Tumor Diseases (NCT), German Cancer Consortium (DKTK), Heidelberg, Germany; ^7^ Takeda Pharma Vertrieb GmbH & Co. KG, Berlin, Germany

**Keywords:** CD19.CAR-T cells, NSAIDs, celecoxib, aspirin, inhibitors, persistence, activation, NF-ĸB pathway

## Abstract

Chimeric antigen receptor T (CAR-T) cells targeting CD19 came into clinical practice for the treatment of B cell lymphoma in 2018. However, patients being treated for B cell lymphoma often suffer from comorbidities such as chronic pain, cardiovascular diseases and arthritis. Thus, these patients frequently receive concomitant medications that include nonsteroidal anti-inflammatory drugs (NSAIDs) like cyclooxygenase (COX) inhibitors. Celecoxib, a selective COX-2 inhibitor, and aspirin, a non-selective COX-1 and COX-2 inhibitor, are being used as anti-inflammatory, analgesic and anti-pyretic drugs. In addition, several studies have also focused on the anti-neoplastic properties of COX-inhibitors. As the influence of COX-inhibitors on CD19.CAR-T cells is still unknown, we investigated the effect of celecoxib and aspirin on the quantity and quality of CD19.CAR-T cells at different concentrations with special regard to cytotoxicity, activation, cytokine release, proliferation and exhaustion. A significant effect on CAR-T cells could be observed for 0.1 mmol/L of celecoxib and for 4 mmol/L of aspirin. At these concentrations, we found that both COX-inhibitors could induce intrinsic apoptosis of CD19.CAR-T cells showing a significant reduction in the ratio of JC-10 red to JC-10 green CAR-T cells from 6.46 ± 7.03 (mean ± SD) to 1.76 ± 0.67 by celecoxib and to 4.41 ± 0.32 by aspirin, respectively. Additionally, the ratios of JC-10 red to JC-10 green Daudi cells were also decreased from 3.41 ± 0.30 to 0.77 ± 0.06 by celecoxib and to 1.26 ± 0.04 by aspirin, respectively. Although the cytokine release by CD19.CAR-T cells upon activation was not hampered by both COX-inhibitors, activation and proliferation of CAR-T cells were significantly inhibited *via* diminishing the NF-ĸB signaling pathway by a significant down-regulation of expression of CD27 on CD4^+^ and CD8^+^ CAR-T cells, followed by a clear decrease of phosphorylated NF-ĸB p65 in both CD4^+^ and CD8^+^ CAR-T cells by a factor of 1.8. Of note, COX-inhibitors hampered expansion and induced exhaustion of CAR-T cells in an antigen stress assay. Collectively, our findings indicate that the use of COX-inhibitors is a double-edged sword that not only induces apoptosis in tumor cells but also impairs the quantity and quality of CAR-T cells. Therefore, COX-inhibitors should be used with caution in patients with B cell lymphoma under CAR-T cell therapy.

## Introduction

Chimeric antigen receptor T (CAR-T) cell therapy is a quantum leap in the treatment of patients with relapsed and/or refractory (r/r) B cell malignancies (1–5). In clinical studies, promising results have been achieved so that the regulatory authorities U.S. Food and Drug Administration (FDA) and European Medicines Agency (EMA) approved two second-generation CD19-directed CAR-T cell products in 2018, i.e., axicabtagene ciloleucel (Yescarta^®^, Kite/Gilead) for the treatment of adult diffuse large B cell lymphoma (DLBCL), transformed follicular lymphoma (tFL) and primary mediastinal B cell lymphoma (PMBCL) as well as tisagenlecleucel (Kymriah^®^, Novartis) for the treatment of pediatric and adolescent acute lymphoblastic leukemia (ALL) and adult DLBCL.

However, patients with r/r B cell lymphoma often suffer from other comorbidities such as chronic pain, cardiovascular diseases, and arthritis, etc. Nonsteroidal anti-inflammatory drugs (NSAIDs) as cyclooxygenase (COX)-inhibitors, celecoxib, a selective COX-2 inhibitor, and aspirin, a non-selective COX-1 and COX-2 inhibitor, are widely being used for the management of these conditions as anti-inflammatory, analgesic and anti-pyretic drugs. Of note, due to their anti-inflammatory effect, the use of COX-inhibitors has been suggested for tumor treatment to suppress the inflammatory tumor microenvironment that promotes neoplastic cell proliferation, survival and migration (6). It might be suggestive for the combination of COX-inhibitors to optimize the CAR-T cell therapy.

In this study, we aimed to investigate the influence of COX-inhibitors on CD19.CAR-T cells added simultaneously as well as after CAR-T cell application with the emphasis on the killing efficiency, activation, cytokine release, proliferation, expansion, and persistence of CD19.CAR-T cells that came in encounter with malignant B cells.

## Materials And Methods

### Cell Lines

CD19^+^ Daudi cells as target cell and CD19^-^ K562 cells as negative control were used in this study, which were purchased from German Collection of Microorganisms and Cell Cultures (DSMZ). Both cell lines were maintained at 5×10^5^ cells/ml in Roswell Park Memorial Institute (RPMI) 1640 medium supplemented with 10% heat-inactivated fetal bovine serum (HI-FBS) according to official guidelines of the DSMZ. Cells were used at logarithmic growth phase and mycoplasma-free in all experiments.

### Isolation of Peripheral Blood Mononuclear Cells (PBMCs)

Buffy coats from healthy donors (HDs) were obtained from the Heidelberg Blood Bank. All participants signed informed consent. PBMCs were separated by a Ficoll gradient (human) (LINARIS), following by freezing them in liquid nitrogen.

### Manufacture of the 3^rd^ Generation CD19.CAR-T Cells

The manufacture of CD19.CAR-T cells mainly includes (a) production of retroviral vector (SFG CD19.CD28/4-1BB/ζ) that was produced by the Good Manufacturing Practice (GMP) Core Facility at Baylor College of Medicine in Houston (7), (b) T cell transduction and (c) CAR-T cell expansion. CD19.CAR-T cells were produced according to our standard operating process (SOPs) as previously described (8–10). The CD19.CAR-T cells from day 10 were used to perform the following experiments.

### CellTiter-Glo Luminescent Cell Viability Assay

To test the cytotoxicity of celecoxib (Medchem express) and aspirin (Sigma Aldrich) on CAR-T cells, CD19.CAR-T cells were seeded into 384-well white wall plates in the presence of 10-point 1:3 serial dilutions of celecoxib and of aspirin starting from 100 μM and 8000 μM, respectively. Then the experiments were performed using the CellTiter Glo reagent (Promega) according to the manufactures’ instructions. After 48-hour co-culture, luminescence was measured by a PerkinElmer plate reader. The cells treated with 0.1% PBS (vehicle) in culture medium for 48 hours were used to normalize the results of experimental group.

### Chromium-51 (^51^Cr) Release Assay

The influence of NSAIDs on the killing efficiency of CD19.CAR-T cells against tumor cells was evaluated by a 4-hour ^51^Cr release assay using an effector to target (E:T) ratio of 30:1. Briefly, effector CD19.CAR-T cells were co-cultured with 5×10^3 51^Cr-labeled tumor cells in the absence or presence of different concentrations of celecoxib or aspirin in triplicates at 37°C with 5% CO_2_ for 4 hours. 75 μl of supernatant per well was collected for the radioactivity readout with a WIZARD^®^Gamma Counter (PerkinElmer). The spontaneous release and maximal release were determined by culturing ^51^Cr-labeled tumor cells with medium and 1% Triton-100 (Sigma-Aldrich), respectively. K562 cells were used as non-specific killing control. The percentage of lysis was calculated as follows: % of lysis = [experimental release - spontaneous release]/[maximal release - spontaneous release] × 100.

### Challenging Assays

To figure out the long-term effect of NSAIDs on CD19.CAR-T cells, challenging assays including simultaneous and subsequent treatment schedules were established in our study. The numbers of residual tumor cells and CD19.CAR-T cells in culture were monitored every five days. Furthermore, use of identical numbers of fresh tumor cells re-challenged CD19.CAR-T cells every five days until no or only a few CAR-T cells were left in the co-culture systems.

#### Simultaneous Treatment Schedule

1.5×10^4^ CD19.CAR-T cells were co-cultured with target Daudi cells at an E:T ratio of 1:1 without or with different concentrations of celecoxib or aspirin in the culture system. Same dosage of celecoxib or aspirin was repeated, when CAR-T cells were re-challenged with the fresh tumor cells.

#### Subsequent Treatment Schedule

Different concentrations of NSAIDs were added into the system, after 24-hour co-culture of CD19.CAR-T cells with target Daudi cells. Repeated addition of the same dose of celecoxib or aspirin was done after 24-hour re-challenge by fresh Daudi cells.

### Flow Cytometry

Multicolor flow cytometry was used to assess the marker expression. Briefly, dead cells were excluded using the Live/Dead Fixable Near-IR Dead Cell Stain Kit (Thermo Fisher Scientific), and then cells were further characterized by staining with different combinations of markers. The antibodies used in this study are shown in **Supplementary Table 1**. All samples were acquired on an LSR II device (BD biosciences) and the flow data were analyzed using Diva (BD biosciences) or FlowJo software (BD biosciences).

#### Activation Marker Staining

CD19.CAR-T cells were stimulated by CD19^+^ Daudi cells for 24-hour at an E:T ratio of 1:1 in the absence or presence of different concentrations of celecoxib or aspirin. Then cells were stained with Near-IR Dead Cell Stain Kit and activation markers in addition with other surface maker antibodies for 30 min at room temperature (RT) in the dark.

#### Cell Viability

For checking the cell viability, Near-IR Dead Cell Stain Kit and Annexin V were used in our study. Briefly, cells were stained with Near-IR Dead Cell Stain Kit for 30 min at 4°C in the dark, followed by surface marker staining (30 min at RT in the dark). Afterwards, cells were washed and re-suspended in Annexin V staining buffer (BD biosciences). Then Annexin V staining (15 min at RT in the dark) was performed to label the apoptotic cells. Acquisition was performed immediately after adding 50 μl of counting beads (Thermo Fisher Scientific). 5,000 counting beads were acquired for each sample.

#### JC-10 Mitochondria Membrane Potential Assay

Mitochondrial membrane potential of cells was measured using JC-10 (Abcam). Firstly, CD19.CAR-T cells and Daudi cells were treated with either celecoxib or aspirin for 2 hours. Then JC-10 staining was performed according to the manufacturer’s instructions. For positive control, cells were stained with JC-10 working solution in the presence of 10 µmol/L carbonyl cyanide 4-(trifluoromethoxy) phenylhydrazone (FCCP) (Sigma Aldrich). Thereafter, cells were fixed by using fixation buffer (Biolegend) prior to analysis.

#### Anti-Apoptotic Bcl-2 Family Protein Staining

Cells co-cultured with Daudi cells (E:T=1:1) in the absence or presence of celecoxib or aspirin for 24 hours, followed by staining with Near-IR Dead Cell Stain Kit. Then the cells were fixed and permeabilized to further stain with anti-apoptotic Bcl-2 family antibody Bcl-xl (30 min at RT in the dark).

#### Intracellular Cytokine Staining

CD19.CAR-T cells were stimulated with CD19^+^ Daudi cells in the presence of monensin (Invitrogen) and brefeldin A (Invitrogen) in addition to either celecoxib or aspirin for 6 hours. After stimulation, the cells were stained with Near-IR Dead Cell Stain Kit (30 min at 4°C in the dark), then fixed, permeabilized and finally stained with surface marker and cytokine antibodies (30 min at RT in the dark).

#### Intracellular Phosphorylated-NF-ĸB p65 (p-NF-ĸB p65) Staining

CD19.CAR-T cells were stimulated with CD19^+^ Daudi cells in the presence of either celecoxib or aspirin for 24 hours. Following the Near-IR Dead Cell Stain Kit staining (30 min at 4°C in the dark), the cells were fixed, permeabilized and stained with surface markers and p-NF-ĸB p65 (invitrogen) (30 min at RT in the dark).

#### Proliferation Assay

5×10^5^ of Carboxy Fluorescein Succinimidyl Ester (CFSE) (BD Horizon™) labeled CD19.CAR-T cells were stimulated with irradiated Daudi cells (Cabinet X-ray Irradiator, X-RAD-320) in a 1:1 ratio for four days in the absence or presence of celecoxib or aspirin. CFSE labeled but non-stimulated CD19.CAR-T cells, stimulated but non-CFSE labeled CD19.CAR-T cells, and Staphylococcal Enterotoxin B (SEB, 1 mg/ml, Sigma-Aldrich) stimulated CD19.CAR-T cells were included as controls in our experiment. After four-day stimulation, the cells were harvested and stained for further analysis.

#### Cell Quantification

After harvest, cells were stained with Near-IR Dead Cell Stain Kit, followed by surface marker staining for both CD19.CAR-T cells and tumor cells (30 min at RT in the dark). Before acquisition 50 μl of counting beads was added immediately. 10,000 counting beads were acquired for each sample to quantify the absolute cell number.

#### Exhaustion Marker Staining

After Near-IR Dead Cell Stain Kit staining, CD19.CAR-T cells were stained with exhaustion markers and other surface maker antibodies for 30 min at RT in the dark.

### Statistical Analysis

IBM SPSS 20 and GraphPad Prism 8.0 were used for statistical analysis. Tukey’s multiple comparisons test and paired t-test were used in comparing the effects of the different concentrations of NSAIDs on CD19.CAR-T cells. Differences were considered statistically significant for *p* value < 0.05.

## Results

### NSAIDs Affect the Quantity and Cytotoxicity of CD19.CAR-T Cells

**Figure 1:**
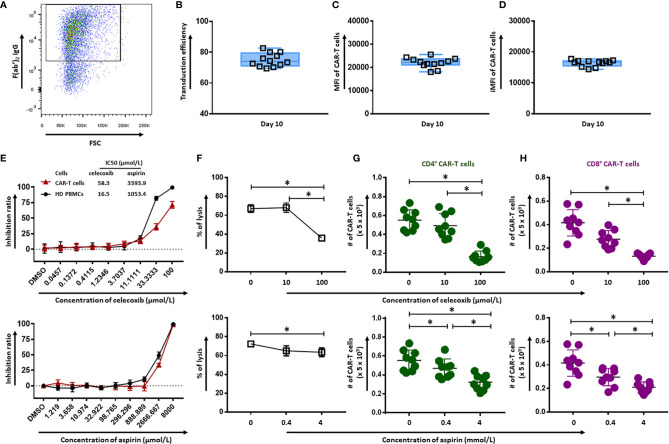
Effect of celecoxib and aspirin on the quantity and cytotoxicity of CD19.CAR-T cells. **(A)** A representative dot plot of the transduction efficiency of CD19.CAR-T cells. Transduction efficiency **(B)**, MFI **(C)** and iMFI **(D)** of CD19.CAR-T cells on day 10. Six batches of manufactured CD19.CAR-T cells from different donors have been analyzed. Each group had two replicates. The inhibition ratios of celecoxib **(E)** (upper panel) and aspirin **(E)** (lower panel) on CD19.CAR-T cells and healthy donor PBMCs. The killing efficiency of CD19.CAR-T cells against Daudi cells in the presence of celecoxib **(F)** (upper panel) and aspirin **(F)** (lower panel) was determined by ^51^Cr-release assay. The living CD4^+^ CAR-T cells **(G)** and CD8^+^ CAR-T cells **(H)** were quantified by flow cytometry after 24 hours co-culture with Daudi cells in the presence of celecoxib (upper panel) and aspirin (lower panel). Data were obtained from three independent experiments. Each experiment had three replicates. A *p* < 0.05 was considered to be statistically significant (*).

CD19.CAR-T cells were successfully generated with a high transduction efficiency (74.88 ± 4.34), as shown in **Figures 1A, B**. The mean fluorescence intensity (MFI) reflecting the CAR molecule expression on a per-cell basis and the integrated MFI (iMFI) indicating the total expression of CAR molecule have been calculated and illustrated in **Figure 1C** (21985 ± 2207) and **Figure 1D** (16389 ± 1036), respectively.

The effect of celecoxib and aspirin on CD19.CAR-T cells and PBMCs from healthy donors was assessed using CellTiter Glo assay. The 50% growth inhibition (IC50) values of celecoxib for CD19.CAR-T cells and HD PBMCs were 58.3 µmol/L and 16.5 µmol/L, respectively, and aspirin with IC50 values of 3393.9 µmol/L and 1053.4 µmol/L, respectively (**Figure 1E**). The cytotoxic activity of celecoxib and aspirin was also tested in a panel of six leukemia and lymphoma cell lines as well as primary chronic lymphocytic leukemia (CLL) cells from ten newly diagnosed and untreated CLL patients (**Supplementary Figure 1**).

To investigate the influence of simultaneous administration of NSAIDs on anti-tumor activity of CD19.CAR-T cells, we cultured CD19.CAR-T cells with tumor cells in the absence or presence of either celecoxib or aspirin. Both NSAIDs impaired cytotoxicity of CD19.CAR-T cells at high doses whereas low doses did not affect the killing efficacy. Especially, celecoxib significantly inhibited the cytotoxic activity of CD19.CAR-T cells at high concentration (**Figure 1F**). Of note, the reduction of the quantity of CD4^+^ and CD8^+^ CD19.CAR-T cells by NSAIDs might contribute to this inhibitory effect on the anti-tumor activity of CAR-T cells (**Figures 1G, H**). The analysis strategy and the representative dot plots were shown in **Supplementary Figures 2** and **3**.

### NSAIDs Induce the Intrinsic Apoptosis Pathway in CD19.CAR-T Cells

**Figure 2:**
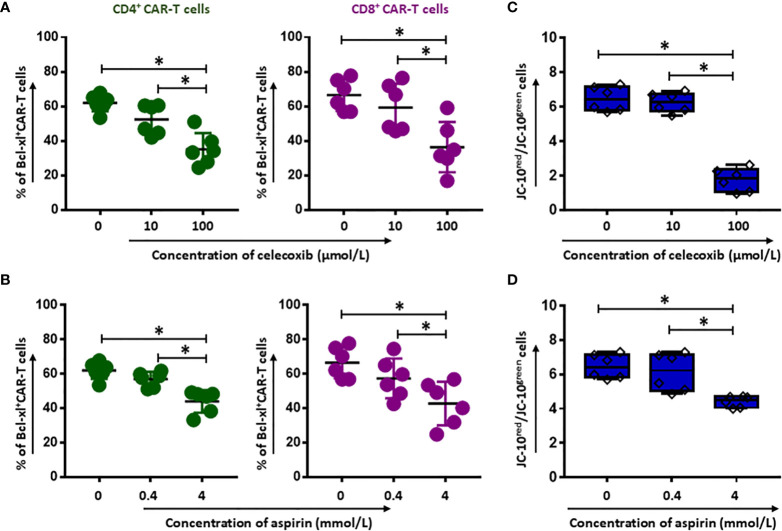
Induction of apoptosis of CD19.CAR-T cells by celecoxib and aspirin. The expression of the anti-apoptotic Bcl-2 family protein Bcl-xl **(A, B)** in the presence of celecoxib (upper panel) and aspirin (lower panel) in CD4^+^ and CD8^+^ CAR-T cells after 24-hour co-culture with Daudi cells. The mitochondrial membrane potential of CD19.CAR-T cells after 2 hours treatment with celecoxib **(C)** and aspirin **(D)**. Data were obtained from three independent experiments. Each experiment had two replicates. A *p* < 0.05 was considered to be statistically significant (*).

To reveal the underlying mechanism of NSAIDs induced CD19.CAR-T cell death, the expression of anti-apoptotic Bcl-2 family protein was determined by intracellular protein staining in CD19.CAR-T cells after 24 hours co-culture with tumor cells in the presence of either celecoxib or aspirin. We observed a dose-dependent decrease of Bcl-xl^+^CD4^+^ (**Figure 2A**) and Bcl-xl^+^CD8^+^ CAR-T cells (**Figure 2B**) for both drugs. Furthermore, our data showed a decreased mitochondrial membrane potential in CD19.CAR-T cells after NSAIDs treatment (**Figures 2C, D**), suggesting that the death of CD19.CAR-T cells might be induced through mitochondria dependent intrinsic apoptosis pathway.

### NSAIDs Impair the Activation and Proliferation Capacity of CD19.CAR-T Cells

**Figure 3:**
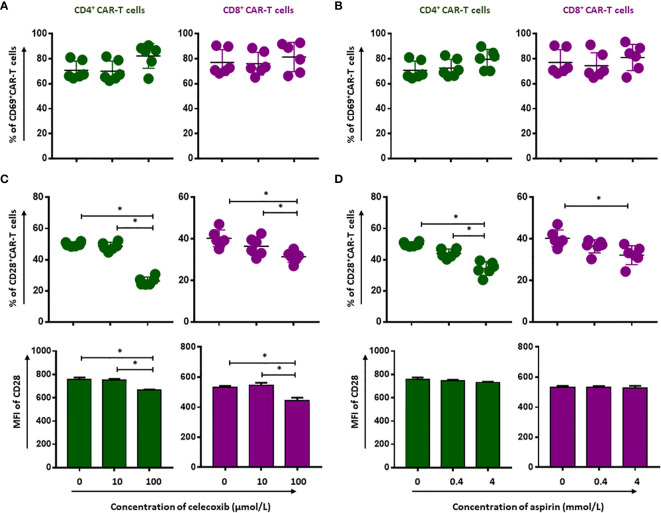
Selective reduction of CD19.CAR-T cell activation by NSAIDs. The expression of activation marker CD69 **(A, B)** and CD28 **(C, D)** on CD4^+^ and CD8^+^ CAR-T cells was assessed using flow cytometry after 24 hours stimulation by Daudi cells in the presence of celecoxib **(A, C)** and aspirin **(B, D)**. Data obtained from three independent experiments. Each experiment had two replicates. A *p* < 0.05 was considered to be statistically significant (*).

**Figure 4:**
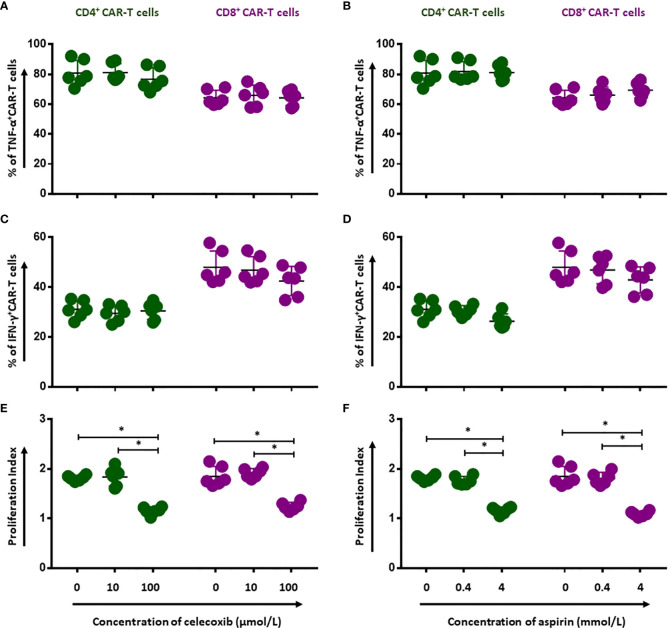
Impairment of proliferation capacity of CD19.CAR-T cells by NSAIDs. Release of TNF-α **(A, B)** and IFN-γ **(C, D)** by CD4^+^ and CD8^+^ CAR-T cells were assessed by flow cytometry after 6-hour stimulation with Daudi cells in the presence of celecoxib **(A, C)** and aspirin **(B, D)**. The proliferation of CD19.CAR-T cells upon activation by irradiated Daudi cells in the presence of celecoxib **(E)** and aspirin **(F)**. Data obtained from three independent experiments. Each experiment had two replicates. A *p* < 0.05 was considered to be statistically significant (*).

To evaluate whether NSAIDs could affect the quality of CD19.CAR-T cells as well, the activation capacity, the cytokine release profile and the proliferation of CD19.CAR-T cells were examined after stimulation by tumor cells in the presence of NSAIDs. We found that both NSAIDs had no significant influence on the expression of the activation marker CD69 (**Figures 3A, B** and **Supplementary Figures 4** and **5A**), the activation marker CD28 however was negatively affected by high doses of NSAIDs (**Figures 3C, D** and **Supplementary Figure 4** and **Supplementary Figure 5B**). Moreover, the TNF-α and IFN-γ release could be maintained by CD19.CAR-T cells upon stimulation (**Figures 4A–D**, **Supplementary Figures 6** and **7A, B**), but the proliferation capacity of both CD4^+^ and CD8^+^ CAR-T cells, which was exhibited in terms of proliferation index (the total number of cell divisions divided by the number of cells that went into division) representing how fast responding cells are proliferating, was hampered by NSAIDs at high doses (**Figures 4E, F** and **Supplementarys Figure 6**, **7C**).

### NSAIDs Hamper the NF-ĸB Pathway *via* Decreasing CD27 and p-NF-ĸB p65 Expression

**Figure 5:**
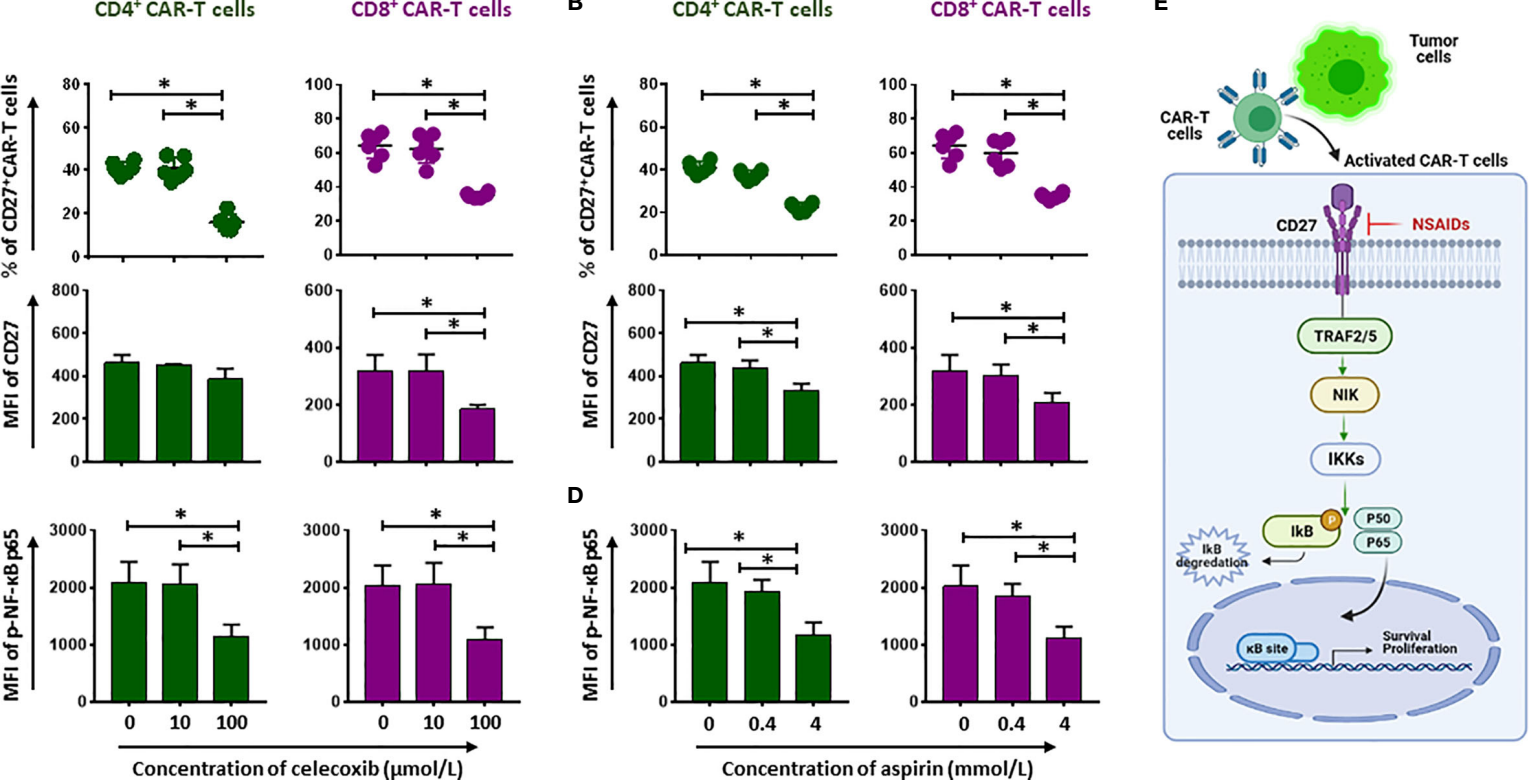
Compromise of CD27-NF-kB signaling pathway by NSAIDs. **(A, B)** show the percentage (upper panel) and MFI (lower panel) expression of CD27 on CD19.CAR-T cells in the presence of celecoxib **(A)** or aspirin **(B)**. The phosphorylated status of NF-ĸB p65 (p-NF-ĸB p65) is presented in **(C, D)**. **(E)** Represents how the NSAIDs affect on the CD27-NF-kB signaling pathway. Data obtained from three independent experiments. Each experiment had two replicates. A *p* < 0.05 was considered to be statistically significant (*).

CD27 is required for generation and long-term maintenance of T cell immunity (11). To better reveal the long-term effects of NSAIDs on CD19.CAR-T cells, we tested CD27 expression after 24-hour treatment with either NSAIDs. We found that CD27 expression on both CD4^+^ and CD8^+^ CD19.CAR-T cells was reduced by high doses of NSAIDs (**Figures 5A, B**, **Supplementary Figures 8**, **9**). CD27 signaling leads to the activation of NF-κB pathway. Thus, we also checked the expression of *p*-NF-κB p65, which reflects the activated status of NF-κB pathway, and observed a decrease of *p*-NF-κB p65 expression (**Figures 5C, D** and **Supplementary Figures 8** and **9**), suggesting that celecoxib and aspirin negatively impact the NF-ĸB signaling pathway (**Figure 5E**).

### NSAIDs Affect the Expansion of CD19.CAR-T Cells

**Figure 6:**
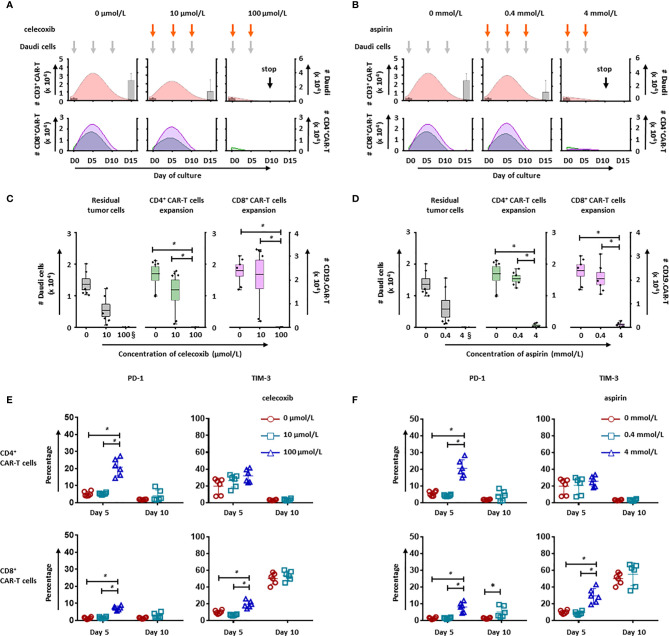
NSAIDs hampered the expansion and persistence of CD19.CAR-T cells in the simultaneous treatment schedule. The influence of celecoxib **(A)** and aspirin **(B)** on dynamics of CD3^+^ CAR-T cells (pink curve charts) and tumor cells (bar chart) were determined by counting beads in a simultaneous treatment schedule. The curves represent the mean value of the absolute number of CD3^+^ CAR-T cells. The CD4^+^ and CD8^+^ CAR-T cells were further analyzed and presented in green curves and violet curves, respectively. The influence of celecoxib **(C)** and aspirin **(D)** on the residual tumor cells on the last day of challenge (day 15), as well as the expansion of CD4^+^ and CD8^+^ CAR-T cells on day 5 were determined by counting beads. **(E, F)** show the expression of exhaustion markers, PD-1 and TIM-3, on CD4^+^ (upper panel) and CD8^+^ CAR-T cells (lower panel) in the presence of celecoxib **(E)** or aspirin **(F)**. § means the number of the residual tumor cells on day 10. Three different individual donors have been analyzed. Each experiment was performed in duplicates. A *p* < 0.05 was considered to be statistically significant (*).

**Figure 7:**
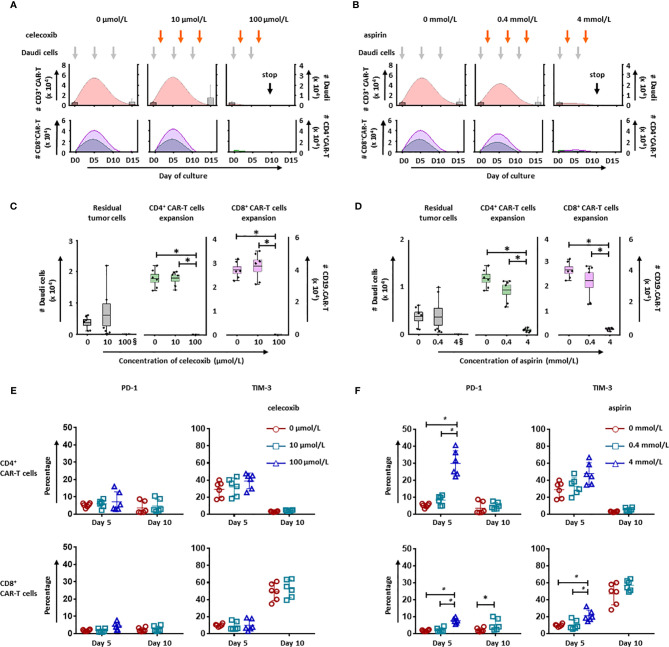
NSAIDs hampered the expansion and persistence of CD19.CAR-T cells in a post treatment schedule. The influence of celecoxib **(A)** and aspirin **(B)** on dynamics of CD3^+^ CAR-T cells (pink curve charts) and tumor cells (bar chart) were determined by counting beads in a post treatment schedule. The curves represent the mean value of the absolute number of CD3^+^ CAR-T cells. The CD4^+^ and CD8^+^ CAR-T cells were further analyzed and presented in green curves and violet curves, respectively. In the presence of celecoxib **(C)** or aspirin **(D)**, the residual tumor cells on the last day of challenge (day 15), as well as the expansion of CD4^+^ and CD8^+^ CAR-T cells on day 5 were represented. § means the number of the residual tumor cells on day 10. The expression of exhaustion markers, PD-1 and TIM-3, on CD4^+^ (upper panel) and CD8^+^ CAR-T cells (lower panel) in the presence of celecoxib **(E)** or aspirin **(F)** were showed. Data obtained from three independent experiments. Each experiment was performed in duplicates. A *p* < 0.05 was considered to be statistically significant (*).

To evaluate the long-term effect of NSAIDs on CD19.CAR-T cells, two different strategies for administration of celecoxib and aspirin were established based on an *in vitro* repetitive challenging assay, including a simultaneous treatment schedule that drugs and tumor cells were introduced to the system simultaneously (**Figure 6**), and a subsequent treatment schedule that drugs were introduced 24 hours after the addition of tumor cells (**Figure 7**). Every five days, CAR-T cells were re-challenged by introducing tumor cells and drugs. Additionally, the number of CAR-T cells and tumor cells, as well as the expression of exhaustion markers (PD-1 and TIM-3) were determined to exhibit the key parameters, including the maximal CD19.CAR-T cell expansion, challenging times, residual tumor cells in the end of coculture, and exhaustion status of CD19.CAR-T cells that could determine the early expansion and persistence of CD19.CAR-T cells.

Upon the activation by CD19^+^ tumor cells, CD19.CAR-T cells extensively proliferated and reached an expansion peak on day 5 in both simultaneous (**Figures 6A, B**) and subsequent treatment schedule (**Figures 7A, B**). However, both drugs could limit the proliferation and expansion of CD19.CAR-T cells at high concentrations in these two treatment schedules. Additionally, the dynamic of CD4^+^ (green curve) and CD8^+^ (violet curve) CAR-T cells showed that the expansion of both cell populations could be negatively affected by celecoxib and aspirin in the treatment systems (**Figures 6A, B**, lower panel), resulting in a very strong reduction of absolute numbers of CD4^+^ and CD8^+^ CD19.CAR-T cells at high doses (**Figures 6C, D**). Moreover, high doses of celecoxib and aspirin could also reduce the maximal challenging times, indicating that CD19.CAR-T cells had an inability to expand. Similar results were observed in the subsequent treatment schedule (**Figure 7**). The analysis strategy and the representative dot plots were shown in **Supplementary Figures 10**, **11A**, **12A**.

### NSAIDs Hamper Persistence of CD19.CAR-T Cells by Inducing Exhaustion

To explore the effect of NSAIDs on the persistence of CD19.CAR-T cells, the expression of exhaustion marker PD-1 and TIM-3 have been monitored on both CD4^+^ and CD8^+^ CAR-T cells in the challenging assay. We found that high doses of celecoxib (100 µmol/L) and aspirin (4 mmol/L) could easily induce an early exhaustion even on day 5, showing an up-regulated PD-1 and TIM-3 expression on both CD4^+^ and CD8^+^ CAR-T cells (**Figures 6E, F**, **Figures 7E, F**, **Supplementary Figures 10**, **11B, C** and **12B, C**). Moreover, low dose of NSAIDs showed a trend towards an increase of PD-1 expression on CD8^+^ CD19.CAR-T cells on day 10 compared to the expression on day 5. Due to the poor proliferation of CD4 subpopulation, similar results were not observed in CD4^+^ CD19.CAR-T cell subsets.

### NSAIDs Induce the Intrinsic Apoptosis Pathway in Daudi Cells

**Figure 8:**
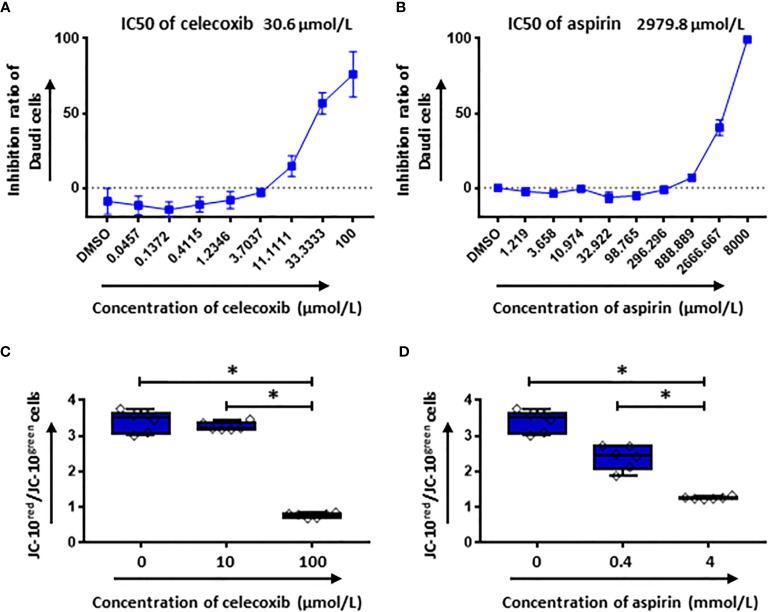
The cytotoxic effect of NSAIDs on Daudi cells is associated with the intrinsic apoptosis pathway. **(A, B)** show the inhibition ratios of celecoxib **(A)** and aspirin **(B)** on Daudi cells. In **(C, D)** the mitochondrial membrane potential of Daudi cells was determined by flow cytometery after 2 hours treatment with celecoxib **(C)** and aspirin **(D)**. Data obtained from three independent experiments. Each experiment had two replicates. A *p* < 0.05 was considered to be statistically significant (*).

Additionally, the cytotoxicity of celecoxib and aspirin on tumor cells was tested using CellTiter Glo assay. Our data showed that both NSAIDs had a dose dependent cytotoxicity on Daudi cells (**Figures 8A, B**). The IC50 of celecoxib was 30.6 µmol/L and aspirin was 2979.8 µmol/L. Moreover, we observed a considerable reduction of mitochondria membrane potential of Daudi cells in the presence of either celecoxib or aspirin at the high concentration (**Figures 8C, D**), indicating that NSAIDs could induce tumor cell death through mitochondria dependent apoptosis pathway.

## Discussion

In recent years the field of adoptive immunotherapy has rapidly advanced to the commercial approvals of genetically engineered T cells expressing chimeric antigen receptors the so-called CAR-T cells for the treatment of B cell hematological malignancies (1, 12–15). CAR-T cells as an innovative and efficient therapy have been successfully applied to critically ill patients. In order not to compromise the effectiveness of the CAR-T cell therapy, it is of great importance that the potency of CAR-T cells *in vivo* is not impaired by concomitant drug therapy. Moreover, manufacturing of CAR-T cells under GMP condition is labor intensive and costly related to quality controls, the one-batch, one-patient paradigm and the complex cold supply chain. Therefore, the definition of potentially CAR-T cell hampering concomitant medications is important from the perspective of patients, clinicians, and also pharmaceutical companies.

NSAIDs, such as celecoxib and aspirin, are widely used medications in managing a number of conditions, including fever, chronic pain, cold or flu, headaches, period pains, joint or bone injuries, sprains and strains, muscle or joint complaints and toothache due to their anti-inflammatory, analgesic and anti-pyretic effects. However, results from previous studies showed inhibitory effects of NSAIDs on normal T cells (16–19). Therefore, the potential influences of NSAIDs on CD19.CAR-T cells raise a concern in the clinicians treating patients with CD19.CAR-T cell therapy and additional NSAIDs. In this study, we aimed to reveal the short-term and long-term effects of celecoxib and aspirin on CD19.CAR-T cells in terms of cytotoxicity, apoptosis, activation, cytokine release, proliferation, expansion, and persistence.

Cytotoxicity against CD19 positive tumor cells is the essential function of CD19.CAR-T cells. A dramatic attenuation of the killing efficacy of CAR-T cell has been observed in our study for both NSAIDs at the highest concentration. The reduction in quantity of CD19.CAR-T cells displaying significant decreases of both CD4^+^ and CD8^+^ CD19.CAR-T cells should be taken into account to this negative effect of NSAIDs on CD19.CAR-T cells. This is might be a consequence of the down-regulation of anti-apoptotic Bcl-2 family members like Bcl-xl in CD19.CAR-T cells, thereby triggering the intrinsic mitochondrial apoptosis pathway in order to induce cell death by apoptosis, which is confirmed by our data and previous studies (17, 19–23).

In addition to the decreased cell number of CD19.CAR-T cells, our results demonstrated that the activation of CD19.CAR-T cells was also affected by NSAIDs. For the activation of third generation CAR-T cells, specific antigens expressed on tumor cells bind to the extracellular CAR domain, a single-chain variable fragment (scFv), and then the signal is transmitted through the transmembrane linker to the intracellular signaling domains such as CD3ζ, CD28 and 4-1BB to induce CAR-T cell activation (24). By NSAIDs treatment, the CD28 molecule that helps to promote CAR-T cell activation was diminished. However, this negative effect on the activation of CAR-T cells might be selective, which is supported by our observation that the activation marker CD69 was not influenced by NSAIDs. This might explain that NSAIDs had no hampering effect on the cytokine release of CD19.CAR-T cells.

After activation, CAR-T cells not only produce cytokines, but also proliferate. Unlike cytokine release, inhibitory effect on the proliferative capacity of CAR-T cells was observed for both NSAIDs. This has been shown by a decrease of the proliferation index that indicates the average number of divisions of all responding cells (25). The CD27-NF-κB pathway plays a crucial role in proliferation (26–28). In our study, the NSAIDs induced reduction of CD27 expression, a member of TNF receptor superfamily, and impaired its binding to TNF receptor associated factors (TRAFs) that link to the NF-κB signaling pathway (29), which leads to blunt the activation of NF-κB pathway. Consequently, NF-κB p65, one of the downstream proteins of activated NF-κB signaling (30), has been less phosphorylated in both CD4^+^ and CD8^+^ CAR-T cells when compared to the group without NSAIDs treatment. The transcription of the downstream encoding genes that are responsible for cellular proliferation could be weakened as a result of hampering phosphorylation of NF-κB p65 (26–28), causing the inhibition of CAR-T cell proliferation.

Apart from mediation of proliferation, CD27-NF-κB pathway is also essential for T cell survival, sustaining effector function, as well as generation and maintenance of long-term functionality of both CD4^+^ and CD8^+^ T cells *via* developing memory T cells (11, 31–33). Considering that the survival of activated T cells could be promoted by CD27 *via* up-regulation of Bcl-xl (34, 35), the decrease of CD27 expression might provide another explanation for the reduction of Bcl-xl expression in NSAIDs-treated CD19.CAR-T cells and contribute to the cell death besides the direct induction of apoptosis by NSAIDs. Moreover, evidence supporting the compromise of the long-term functionality of CD19.CAR-T cells by NSAIDs has been derived from an antigen stress test, so called challenging assay. Our results indicate that both NSAID medications have negative effects not only on the expansion of CD19.CAR-T cells, but also on their repetitive killing capacity. Additionally, CAR-T cells were prone to exhaustion by administration of NASIDs, which is supported by our observation showing an up-regulation of exhaustion markers PD-1 and TIM-3. The low proliferation rate and limited anti-tumor efficacy further confirmed this exhausted status in the functional level. Normally, T cell exhaustion is a state of T cell dysfunction that arises during persistent antigen exposure (36). However, it seems not be the case in our study, since celecoxib and aspirin could directly induce the apoptosis and display an anti-proliferative effect on tumor cells. Therefore, the acceleration of exhaustion of CAR-T cells might be also a consequence of the impairment of the CD27-NF-κB pathway by NSAIDs.

According to our data relevant doses of NSAIDs for hampering significantly CAR-T cells have been 4 mmol/L for aspirin and 0.1 mmol/L for celecoxib in *in vitro* culture systems. Reviewing the literature, a mean peak level for aspirin of 6.5 µg/mL (0.036 mmol/L) (range 4.9-8.9 µg/ml, 0.027-0.049 mmol/L) and for salycilate of 49 µg/mL (0.36 mmol/L) (range 42-62 µg/mL, 0.31-0.45 mmol/L) was reached in plasma after a single oral dose of 975 mg (37, 38). So, aspirin often taken prophylactically at a much lower dose of 100 mg/d might not reach relevant plasma levels to hamper CAR-T cells. For celecoxib however mean maximum plasma levels of 1.53 mg/ml (4 mmol/L) were measured after a dose of 300 mg (39). Doses of 200 mg per day are recommended for analgetic, anti-inflammatory indications and even higher doses for tumor therapy (40, 41). So, celecoxib plasma levels even used at lower doses might be far above the relevant CAR-T cell hampering dose of 0.1 mmol/L. Therefore, NSAIDs in particular celecoxib should be used with caution simultaneously with or after CAR-T cell administration in patients.

In summary, although celecoxib and aspirin display an anti-tumor effect, the quantity and quality of CD19.CAR-T cells could be hampered in a dose dependent manner. Relevant CAR-T cell hampering might be reached by celecoxib therapy. Therefore, our study provides the rationale that celecoxib and CAR-T cell therapy should be used together with caution, and discovers the potential mechanisms that NSAIDs negatively affect on CD19.CAR-T cells through their effects on the induction of apoptosis, reduction of activation and impairment of CD27-NF-κB pathway.

## Data Availability Statement

The raw data supporting the conclusions of this article will be made available by the authors, without undue reservation.

## Ethics Statement

Sample collection and analysis were approved by the Ethics Committee of the University of Heidelberg (S-254/2016). The patients/participants provided their written informed consent to participate in this study.

## Author Contributions

AS, MS, LW, and MY designed the research. MY performed the experiments. SMW, WJG collected and stored the samples. MY and LW analyzed the data. MS, AS, LW, MY, MN, BN, TS, M-LS, AH-K, JG, LS, CK, and VE discussed the organization of the manuscript. LW and MY wrote the manuscript. All authors critically reviewed the manuscript. MS, AS, CM-T, RX, and PD edited the manuscript. AS and LW supervised the work. All authors contributed to the article and approved the submitted version.

## Funding

This research was funded by Deutsche Forschungsgemeinschaft within the funding program Open Access Publishing, by the Baden-Württemberg Ministry of Science, Research and the Arts and by Ruprecht-Karls-Universität Heidelberg. MY is supported by a scholarship from China Scholarship Council.

## Conflict of Interest

MS received funding for collaborative research from Apogenix, Hexal and Novartis, travel grants from Hexal and Kite, he received financial support for educational activities and conferences from bluebird bio, Kite and Novartis, he is a board member for MSD and (co-)PI of clinical trials of MSD, GSK, Kite and BMS, as well as co-founder and shareholder of TolerogenixX Ltd. AS received travel grants from Hexal and Jazz Pharmaceuticals, research grant from Therakos/Mallinckrodt and is co-founder of TolerogenixX Ltd. AS and LW are part- or full-time employers of TolerogenixX Ltd. LS was employed by Takeda Pharma Vertrieb GmbH & Co. KG.

The remaining authors declare that the research was conducted in the absence of any commercial or financial relationships that could be construed as a potential conflict of interest.

## References

[1] Maude SL Laetsch TW Buechner J Rives S Boyer M Bittencourt H. Tisagenlecleucel in Children and Young Adults With B-Cell Lymphoblastic Leukemia. N Engl J Med (2018) 378(5):439–48. 10.1056/NEJMoa1709866 PMC5996391 29385370

[2] Turtle CJ Hay KA Hanafi LA Li D Cherian S Chen X. Durable Molecular Remissions in Chronic Lymphocytic Leukemia Treated With Cd19-Specific Chimeric Antigen Receptor-Modified T Cells After Failure of Ibrutinib. J Clin Oncol Off J Am Soc Clin Oncol (2017) 35(26):3010–20. 10.1200/JCO.2017.72.8519 PMC5590803 28715249

[3] Kochenderfer JN Somerville RPT Lu T Yang JC Sherry RM Feldman SA. Long-Duration Complete Remissions of Diffuse Large B Cell Lymphoma After Anti-CD19 Chimeric Antigen Receptor T Cell Therapy. Mol Ther J Am Soc Gene Ther (2017) 25(10):2245–53. 10.1016/j.ymthe.2017.07.004 PMC5628864 28803861

[4] Neelapu SS Locke FL Bartlett NL Lekakis LJ Miklos DB Jacobson CA. Axicabtagene Ciloleucel Car T-Cell Therapy in Refractory Large B-Cell Lymphoma. N Engl J Med (2017) 377(26):2531–44. 10.1056/NEJMoa1707447 PMC5882485 29226797

[5] Schuster SJ Svoboda J Chong EA Nasta SD Mato AR Anak Ö. Chimeric Antigen Receptor T Cells in Refractory B-Cell Lymphomas. N Engl J Med (2017) 377(26):2545–54. 10.1056/NEJMoa1708566 PMC5788566 29226764

[6] Tołoczko-Iwaniuk N Dziemiańczyk-Pakieła D Nowaszewska BK Celińska-Janowicz K Miltyk W. Celecoxib in Cancer Therapy and Prevention - Review. Curr Drug Targets (2019) 20(3):302–15. 10.2174/1389450119666180803121737 30073924

[7] Enblad G Karlsson H Gammelgard G Wenthe J Lovgren T Amini RM. A Phase I/Iia Trial Using CD19-Targeted Third-Generation Car T Cells for Lymphoma and Leukemia. Clin Cancer Res (2018) 24(24):6185–94. 10.1158/1078-0432.CCR-18-0426 30097433

[8] Wang L Gong W Wang S Neuber B Sellner L Schubert ML. Improvement of In Vitro Potency Assays by a Resting Step for Clinical-Grade Chimeric Antigen Receptor Engineered T Cells. Cytotherapy (2019) 21(5):566–78. 10.1016/j.jcyt.2019.02.013 30910382

[9] Hoffmann JM Schubert ML Wang L Huckelhoven A Sellner L Stock S. Differences in Expansion Potential of Naive Chimeric Antigen Receptor T Cells From Healthy Donors and Untreated Chronic Lymphocytic Leukemia Patients. Front Immunol (2017) 8:1956. 10.3389/fimmu.2017.01956 29375575 PMC5767585

[10] Stock S Hoffmann JM Schubert ML Wang L Wang S Gong W. Influence of Retronectin-Mediated T-Cell Activation on Expansion and Phenotype of CD19-Specific Chimeric Antigen Receptor T Cells. Hum Gene Ther (2018) 29(10):1167–82. 10.1089/hum.2017.237 30024314

[11] Hendriks J Gravestein LA Tesselaar K van Lier RA Schumacher TN Borst J. CD27 is Required for Generation and Long-Term Maintenance of T Cell Immunity. Nat Immunol (2000) 1(5):433–40. 10.1038/80877 11062504

[12] Labanieh L Majzner RG Mackall CL. Programming CAR-T Cells to Kill Cancer. Nat BioMed Eng (2018) 2(6):377–91. 10.1038/s41551-018-0235-9 31011197

[13] Maude SL Frey N Shaw PA Aplenc R Barrett DM Bunin NJ. Chimeric Antigen Receptor T Cells for Sustained Remissions in Leukemia. N Engl J Med (2014) 371(16):1507–17. 10.1056/NEJMoa1407222 PMC4267531 25317870

[14] Lee DW Kochenderfer JN Stetler-Stevenson M Cui YK Delbrook C Feldman SA. T Cells Expressing CD19 Chimeric Antigen Receptors for Acute Lymphoblastic Leukaemia in Children and Young Adults: A Phase 1 Dose-Escalation Trial. Lancet (2015) 385(9967):517–28. 10.1016/S0140-6736(14)61403-3 PMC7065359 25319501

[15] Park JH Riviere I Gonen M Wang X Senechal B Curran KJ. Long-Term Follow-up of CD19 Car Therapy in Acute Lymphoblastic Leukemia. N Engl J Med (2018) 378(5):449–59. 10.1056/NEJMoa1709919 PMC6637939 29385376

[16] Gao J Niwa K Sun W Takemura M Lian Z Onogi K. Non-Steroidal Anti-Inflammatory Drugs Inhibit Cellular Proliferation and Upregulate Cyclooxygenase-2 Protein Expression in Endometrial Cancer Cells. Cancer sci (2004) 95(11):901–7. 10.1111/j.1349-7006.2004.tb02200.x PMC11159916 15546508

[17] Iniguez MA Punzon C Fresno M. Induction of Cyclooxygenase-2 on Activated T Lymphocytes: Regulation of T Cell Activation by Cyclooxygenase-2 Inhibitors. J Immunol (1999) 163(1):111–9. 10.1056/NEJMoa1707447 10384106

[18] Paccani SR Boncristiano M Ulivieri C D’Elios MM Del Prete G Baldari CT. Nonsteroidal Anti-Inflammatory Drugs Suppress T-cell Activation by Inhibiting P38 MAPK Induction. J Biol Chem (2002) 277(2):1509–13. 10.1074/jbc.M110676200 11700329

[19] Yin MJ Yamamoto Y Gaynor RB. The Anti-Inflammatory Agents Aspirin and Salicylate Inhibit the Activity of I(kappa)B Kinase-Beta. Nature (1998) 396(6706):77–80. 10.1038/23948 9817203

[20] Hossain MA Kim DH Jang JY Kang YJ Yoon JH Moon JO. Aspirin Induces Apoptosis In Vitro and Inhibits Tumor Growth of Human Hepatocellular Carcinoma Cells in a Nude Mouse Xenograft Model. Int J Oncol (2012) 40(4):1298–304. 10.3892/ijo.2011.1304 PMC3584583 22179060

[21] Jana NR. Nsaids and Apoptosis. Cell Mol Life Sci (2008) 65(9):1295–301. 10.1007/s00018-008-7511-x PMC11131660 18292966

[22] Jin M Li C Zhang Q Xing S Kan X Wang J. Effects of Aspirin on Proliferation, Invasion and Apoptosis of Hep-2 Cells Via the PTEN/AKT/NF-κb/Survivin Signaling Pathway. Oncol lett (2018) 15(6):8454–60. 10.3892/ol.2018.8377 PMC5950550 29805582

[23] Rudner J Elsaesser SJ Müller AC Belka C Jendrossek V. Differential Effects of Anti-Apoptotic Bcl-2 Family Members Mcl-1, Bcl-2, and Bcl-xL on Celecoxib-Induced Apoptosis. Biochem Pharmacol (2010) 79(1):10–20. 10.1016/j.bcp.2009.07.021 19665451

[24] Hofmann S Schubert ML Wang L He B Neuber B Dreger P. Chimeric Antigen Receptor (Car) T Cell Therapy in Acute Myeloid Leukemia (Aml). J Clin Med (2019) 8(2):200. 10.3390/jcm8020200 PMC6406805 30736352

[25] Roederer M. Interpretation of Cellular Proliferation Data: Avoid the Panglossian. Cytometry A (2011) 79(2):95–101. 10.1002/cyto.a.21010 21265003

[26] Baldwin AS. Regulation of Cell Death and Autophagy by IKK and NF-kappaB: Critical Mechanisms in Immune Function and Cancer. Immunol Rev (2012) 246(1):327–45. 10.1111/j.1600-065X.2012.01095.x 22435564

[27] Basak S Behar M Hoffmann A. Lessons From Mathematically Modeling the NF-kappaB Pathway. Immunol Rev (2012) 246(1):221–38. 10.1111/j.1600-065X.2011.01092.x PMC3343698 22435558

[28] Yde P Mengel B Jensen MH Krishna S Trusina A. Modeling the NF-kappaB Mediated Inflammatory Response Predicts Cytokine Waves in Tissue. BMC Syst Biol (2011) 5:115. 10.1186/1752-0509-5-115 21771307 PMC3152534

[29] Aggarwal BB. Signalling Pathways of the TNF Superfamily: A Double-Edged Sword. Nat Rev Immunol (2003) 3(9):745–56. 10.1038/nri1184 12949498

[30] Giridharan S Srinivasan M. Mechanisms of NF-kappaB p65 and Strategies for Therapeutic Manipulation. J Inflammation Res (2018) 11:407–19. 10.2147/JIR.S140188 PMC6217131 30464573

[31] Hendriks J Xiao Y Borst J. CD27 Promotes Survival of Activated T Cells and Complements CD28 in Generation and Establishment of the Effector T Cell Pool. J Exp Med (2003) 198(9):1369–80. 10.1084/jem.20030916 PMC2194245 14581610

[32] van de Ven K Borst J. Targeting the T-cell Co-Stimulatory CD27/CD70 Pathway in Cancer Immunotherapy: Rationale and Potential. Immunotherapy (2015) 7(6):655–67. 10.2217/imt.15.32 26098609

[33] Zhang L Blackwell K Altaeva A Shi Z Habelhah H. TRAF2 Phosphorylation Promotes NF-κb-Dependent Gene Expression and Inhibits Oxidative Stress-Induced Cell Death. Mol Biol Cell (2011) 22(1):128–40. 10.1091/mbc.e10-06-0556 PMC3016971 21119000

[34] Peperzak V Veraar EA Keller AM Xiao Y Borst J. The Pim Kinase Pathway Contributes to Survival Signaling in Primed CD8+ T Cells Upon CD27 Costimulation. J Immunol (2010) 185(11):6670–8. 10.4049/jimmunol.1000159 21048108

[35] van Oosterwijk MF Juwana H Arens R Tesselaar K van Oers MH Eldering E. Cd27-CD70 Interactions Sensitise Naive CD4+ T Cells for IL-12-induced Th1 Cell Development. Int Immunol (2007) 19(6):713–8. 10.1093/intimm/dxm033 17548342

[36] Wherry EJ. T Cell Exhaustion. Nat Immunol (2011) 12(6):492–9. 10.1038/ni.2035 21739672

[37] Kershaw RA Mays DC Bianchine JR Gerber N. Disposition of Aspirin and its Metabolites in the Semen of Man. J Clin Pharmacol (1987) 27(4):304–9. 10.1002/j.1552-4604.1987.tb03019.x 3680588

[38] Kees F Jehnich D Grobecker H. Simultaneous Determination of Acetylsalicylic Acid and Salicylic Acid in Human Plasma by High-Performance Liquid Chromatography. J Chromatogr B: Biomed Sci Appl (1996) 677(1):172–7. 10.1016/0378-4347(95)00464-5 8925092

[39] Paulson SK Hribar JD Liu NW Hajdu E Bible RH Jr. Piergies A. Metabolism and Excretion of [(14)C]Celecoxib in Healthy Male Volunteers. Drug Metab Dispos (2000) 28(3):308–14. 10681375

[40] Li S Jiang M Wang L Yu S. Combined Chemotherapy With Cyclooxygenase-2 (COX-2) Inhibitors in Treating Human Cancers: Recent Advancement. Biomed pharmacother = Biomedecine pharmacotherapie (2020) 129:110389. 10.1016/j.biopha.2020.110389 32540642

[41] Steinbach G Lynch PM Phillips RK Wallace MH Hawk E Gordon GB. The Effect of Celecoxib, a Cyclooxygenase-2 Inhibitor, in Familial Adenomatous Polyposis. N Engl J Med (2000) 342(26):1946–52. 10.1056/NEJM200006293422603 10874062

