# Supplementary material for: Dual Effects of Cyclooxygenase Inhibitors in Combination With CD19.CAR-T Cell Immunotherapy
Source: Front Immunol. 2021 May 26;12:670088. doi: 10.3389/fimmu.2021.670088 (PMC8189155; doi:10.3389/fimmu.2021.670088)
Supplement: Supplementary file 1 [file DataSheet_1.docx]

Supplementary Material

Dual effects of cyclooxygenase inhibitors in combination with CD19.CAR-T cell immunotherapy

Mingya Yang^1,2†^, Lei Wang^1†^, Ming Ni^1,3^, Brigitte Neuber^1^, Sanmei Wang^1^, Wenjie Gong^1,4,^ Tim Sauer^1^, Maria-Luisa Schubert^1^, Angela Hückelhoven-Krauss^1^, Ruixiang Xia^2^, Jian Ge^2^, Christian Kleist^5^, Volker Eckstein^1^, Leopold Sellner^1,§^, Carsten Müller-Tidow^1,6^, Peter Dreger^1,6^, Michael Schmitt^1,6,^ Anita Schmitt^1^

^1^Department of Internal Medicine V, University Clinic Heidelberg, Heidelberg University, Heidelberg, Germany

^2^Department of Hematology, the First Affiliated Hospital of Anhui Medical University, Anhui, China

^3^Department of Hematology, the Affiliated Hospital of Guizhou Medical University, Guizhou, China

^4^Department of Hematology, the first Affiliated Hospital of Soochow University, Suzhou, China

^5^Department of Nuclear Medicine, University Clinic Heidelberg, Heidelberg University, Heidelberg, Germany

^6^National Center for Tumor Diseases (NCT), German Cancer Consortium (DKTK), Heidelberg, Germany

^§^Takeda Pharma Vertrieb GmbH & Co. KG, Berlin, Germany

^†^These authors have contributed equally to this work and share first authorship.

***Correspondence:**PD Dr. med. Anita Schmitt
anita.schmitt@med.uni-heidelberg.de

# Supplementary Table 1. Antibody list.

| **Antibody** | **Dye** | **Isotype** | **Clone** | **V_working_ (μl)** | **Company** | **Cat.No.** |
| --- | --- | --- | --- | --- | --- | --- |
| Bcl-xl | PerCP | IgG2a | 2H12 | 1 | NOVUS | NBP2-34531PCP |
| CD10 | PE-Cy7 | Mouse IgG1, k | HI10a | 1 | Biolegend | 312214 |
| CD28 | FITC | Mouse IgG1, κ | CD28.2 | 2 | BD Biosciences | 555728 |
| CD27 | PE-Cy7 | Mouse IgG1, k | M-T271 | 1 | Biolegend | 356412 |
| CD3 | PE-Cy7 | Mouse IgG1, k | UCHT1 | 1 | Biolegend | 300420 |
| CD3 | BV510 | Mouse IgG2a, k | OKT3 | 1 | Biolegend | 317332 |
| CD3 | FITC | Mouse IgG1, k | UCHT1 | 1 | Biolegend | 300440 |
| CD4 | PE-CF594 | Mouse IgG1, k | RPA-T4 | 1 | BD Biosciences | 562281 |
| CD4 | APC | Mouse IgG1, k | SK3 | 1 | Biolegend | 344614 |
| CD69 | APC | Mouse IgG1, k | FN50 | 1 | Biolegend | 310910 |
| CD8 | BV510 | Mouse BALB/c IgG1,k | SK1 | 1 | BD Biosciences | 563919 |
| CD8 | APC | Mouse IgG1, k | SK1 | 1 | Biolegend | 344722 |
| CD8 | PerCP | Mouse IgG1, k | SK1 | 0.5 | Biolegend | 344708 |
| gt F(ab’)2 IgG | PE | Ig | --- | 0.5 | Dianova | 109-116-088 |
| IFN-γ | APC | Mouse IgG1, k | B27 | 1 | eBioscience | 554702 |
| Phospho- κB-p65 antibody | PE | Mouse IgG1, k | B33B4WP | 1 | Invitrogen | 12-9863-42 |
| PD-1 | Alexa Fluor 488 | Mouse IgG1, k | EH12.2H7 | 1 | Biolegend | 329936 |
| Tim-3 | BV421 | Mouse IgG1, k | F38-2E2 | 1 | Biolegend | 345008 |
| TNF-α | BV421 | Mouse IgG1, k | MAb11 | 1 | eBioscience | 562783 |

Abbreviations: V_working_ = volume of the antibody per 100 μl cocktail solution; Cat.No. = catalogue number; PE = phycoerythrin; PE-Cy7 = phycoerythrin-Cyanin 7; BV = Brilliant Violet; PerCP = peridinin chlorophyll A; APC = allophycocyanin; IFN-γ = Interferon-γ; TNF-α = Tumor necrosis factor-α; FITC = Fluorescein isothiocyanate; Bcl-xl = B-cell lymphoma-extra-large; PD-1 = Programmed cell death protein 1; TIM-3 = T-cell immunoglobulin and mucin-domain containing-3

# Supplementary Figure Legends

## Supplementary Figure 1. Inhibitory effect of celecoxib and aspirin on tumor cells

The inhibition ratios of celecoxib and aspirin on different cells after 24-hour incubation, which were calculated according to the formula: inhibition ratio = 100 – (experimental luminescence/PBS luminescence) × 100. IC50 was determined by IBM SPSS v22, regression-probit. Data were obtained from three independent experiments.

## Supplementary Figure 2. Analysis strategy of panels of apoptosis and anti-apoptotic protein Bcl-xl

Representative dot plots of analysis strategy of apoptosis and intracellular staining of Bcl-xl in CD19.CAR-T cells. Dead cells were excluded by Near-IR before the subsequent analysis. The residual tumor cells were excluded by CD3 antibody, since Daudi cells were CD3^-^. 5,000 counting beads were acquired to quantify the number of events. Fluorescence Minus One (FMO) controls were used to gate the populations of Annexin V^-^ and Bcl-xl^+^ CAR-T cells, respectively.

## Supplementary Figure 3. Representative dot plots of apoptosis and expression of anti-apoptotic protein Bcl-xl in CD4^+^ and CD8^+^ CAR-T cells

A set of representative dot plots of the counts of residual living CD4^+^ and CD8^+^ CAR-T cells (Annexin V^-^) (A), and the percentage of Bcl-xl^+^ CAR-T cells (B) in the CD4^+^ and CD8^+^ subpopulations in the presence of celecoxib and aspirin after 24-hour co-culture with Daudi cells.

## Supplementary Figure 4. Analysis strategy of activation panel

Representative dot plots of analysis strategy of activation marker expression on CD19.CAR-T cells. Dead cells were excluded by Near-IR before the subsequent analysis. The residual tumor cells were excluded by CD3 antibody, because Daudi cells were CD3^-^. FMO controls were used to gate the populations of CD69^+^ and CD28^+^ CAR-T cells, respectively.

## Supplementary Figure 5. Representative dot plots of expression of activation markers on CD4^+^ and CD8^+^ CAR-T cells

A set of representative dot plots of the percentage of CD69^+^ (A) and CD28^+^ (B) CAR-T cells in the CD4^+^ and CD8^+^ subpopulations in the presence of celecoxib and aspirin after 24-hour co-culture with Daudi cells.

## Supplementary Figure 6. Analysis strategy of panels of cytokine release and proliferation

Representative plots of analysis strategy of proliferation and cytokine release of CD19.CAR-T cells. Dead cells were excluded by Near-IR before the subsequent analysis. The residual tumor cells were excluded by CD3 antibody, because Daudi cells were CD3^-^. Proliferation index was calculated by flowJo-proliferation platform. CD19^-^ K562 cells stimulated CD19.CAR-T cell group was used to set gates of TNF-α^+^ and IFN-γ^+^ CAR-T cells, respectively.

## Supplementary Figure 7. Representative dot plots of cytokine release and proliferation of CD4^+^ and CD8^+^ CAR-T cells

A set of representative dot plots of the percentage of TNF-α^+^ (A) and IFN-γ^+^ (B) CAR-T cells as well as of the histogram of proliferation of CFSE-labeled (C) in the CD4^+^ and CD8^+^ CAR-T cells cocultured with Daudi cells in the presence of celecoxib and aspirin. Treatment durations of cytokine release and proliferation assays were 6 hours and 4 days, respectively.

## Supplementary Figure 8. Analysis strategy of panels of CD27 and phosphorylated-NF-ĸB p65

Representative plots of analysis strategy of expression of CD27 and phosphorylated-NF-ĸB p65 in CD19.CAR-T cells. Dead cells were excluded by Near-IR before the subsequent analysis. The residual tumor cells were excluded by CD3 antibody, because Daudi cells were CD3^-^. FMO control was used to set the gate of CD27^+^ CAR-T cells. Histogram of phosphorylated-NF-ĸB p65 was showed to reflect the protein intensity in CAR-T cells.

## Supplementary Figure 9. Representative plots of CD27 and phosphorylated-NF-ĸB p65 expression in CD4^+^ and CD8^+^ CAR-T cells

A set of representative dot plots of the percentage of CD27^+^ CAR-T cells (left panel) and histograms of phosphorylated-NF-ĸB p65 in the CD4^+^ and CD8^+^ subpopulations (right panel) in the presence of celecoxib (A) and aspirin (B) after 24-hour co-culture with Daudi cells.

## Supplementary Figure 10. Analysis strategy of panels of quantification of tumor cells and CAR-T cells as well as exhaustion markers expression

Representative dot plots of analysis strategy of quantification of Daudi and CAR-T cells, as well as the expression of exhaustion markers, PD-1 and TIM-3, on CD4^+^ and CD8^+^ CAR-T cells. Dead cells were excluded by Near-IR before the subsequent analysis. 10,000 counting beads were acquired to quantify the cell number. The residual tumor cells were gated out by CD10 antibody, because Daudi cells were CD10^+^. FMO controls were used to set the gate of PD-1^+^ and TIM-3^+^ CAR-T cells.

## Supplementary Figure 11. Representative plots of the residual Daudi and CAR-T cells and exhaustion markers expression on CD4^+^ and CD8^+^ CAR-T cells in the simultaneous treatment schedule

A set of representative dot plots of the counts of Daudi (CD10^+^CD3^-^) and T cells (CD3^+^CD10^-^) (**A**) as well as percentages of PD-1^+^ (**B**) and TIM-3^+^ (**C**) CAR-T cells in the CD4^+^ and CD8^+^ subpopulations on day 5 in the presence of celecoxib and aspirin in the simultaneous treatment schedule.

## Supplementary Figure 12. Representative plots of the residual Daudi and CAR-T cells and exhaustion markers expression on CD4^+^ and CD8^+^ CAR-T cells in the post treatment schedule

A set of representative dot plots of the counts of Daudi (CD10^+^CD3^-^) and T cells (CD3^+^CD10^-^) (**A**) as well as percentages of PD-1^+^ (**B**) and TIM-3^+^ (**C**) CAR-T cells in the CD4^+^ and CD8^+^ subpopulations on day 5 in the presence of celecoxib and aspirin in the post treatment schedule.
